# Development and validation of risk prediction models for multiple cardiovascular diseases and Type 2 diabetes

**DOI:** 10.1371/journal.pone.0235758

**Published:** 2020-07-29

**Authors:** Mehrdad Rezaee, Igor Putrenko, Arsia Takeh, Andrea Ganna, Erik Ingelsson

**Affiliations:** 1 Mynomx Inc., Palo Alto, CA, United States of America; 2 Cardiac and Vascular Care Inc., San Jose, CA, United States of America; 3 Program in Medical and Population Genetics, Broad Institute of MIT and Harvard, Cambridge, MA, United States of America; 4 Stanley Center for Psychiatric Research, Broad Institute of MIT and Harvard, Cambridge, MA, United States of America; 5 Analytic and Translational Genetics Unit, Department of Medicine, Massachusetts General Hospital, Boston, MA, United States of America; 6 Division of Cardiovascular Medicine, Department of Medicine, Stanford University School of Medicine, Stanford, CA, United States of America; 7 Stanford Cardiovascular Institute, Stanford, CA, United States of America; 8 Stanford Diabetes Research Center, Stanford, CA, United States of America; Stellenbosch University Faculty of Medicine and Health Sciences, SOUTH AFRICA

## Abstract

Accurate risk assessment of an individuals’ propensity to develop cardiovascular diseases (CVDs) is crucial for the prevention of these conditions. Numerous published risk prediction models used for CVD risk assessment are based on conventional risk factors and include only a limited number of biomarkers. The addition of novel biomarkers can boost the discriminative ability of risk prediction models for CVDs with different pathogenesis. The present study reports the development of risk prediction models for a range of heterogeneous CVDs, including coronary artery disease (CAD), stroke, deep vein thrombosis (DVT), and abdominal aortic aneurysm (AAA), as well as for Type 2 diabetes mellitus (DM2), a major CVD risk factor. In addition to conventional risk factors, the models incorporate various blood biomarkers and comorbidities to improve both individual and population stratification. An automatic variable selection approach was developed to generate the best set of explanatory variables for each model from the initial panel of risk factors. In total, up to 254,220 UK Biobank participants (ranging from 215,269 to 254,220 for different CVDs and DM2) were included in the analyses. The derived prediction models utilizing Cox proportional hazards regression achieved consistent discrimination performance (C-index) for all diseases: CAD, 0.794 (95% CI, 0.787–0.801); DM2, 0.909 (95% CI, 0.903–0.916); stroke, 0.778 (95% CI, 0.756–0.801); DVT, 0.743 (95% CI, 0.737–0.749); and AAA, 0.893 (95% CI, 0.874–0.912). When validated on various subpopulations, they demonstrated higher discrimination in healthier and middle-age individuals. In general, calibration of a five-year risk of developing the CVDs and DM2 demonstrated incremental overestimation of disease-related conditions amongst the highest decile of risk probabilities. In summary, the risk prediction models described were validated with high discrimination and good calibration for several CVDs and DM2. These models incorporate multiple shared predictor variables and may be integrated into a single platform to enhance clinical stratification to impact health outcomes.

## Introduction

Cardiovascular diseases (CVDs) include a range of chronic diseases that impair cardiac and vascular function, which continues to be the leading cause of death in the United States (US). It is projected that over 45% of the US population will suffer from one or more CVDs by 2035 [1]. The healthcare cost associated with CVDs represents one of the greatest global economic burdens [2].

As with any chronic condition, appropriate prevention and selective treatment of CVDs are the most effective approaches to reduce their clinical and financial impact. Accurate risk assessment of an individual’s propensity to develop CVDs is essential for personalized health care and primary prevention of these conditions. An increasing number of novel biomarkers have been linked to CVD risk [3–14], implying their critical role in precise risk assessment for heterogeneous CVDs. Current established functions for CVD risk stratification are either based only on conventional risk factors or include a limited number of biomarkers [15–18]. Furthermore, the contribution of various biomarkers to the risk of CVDs with different pathogenesis is poorly understood.

In this study, we sought to improve CVD risk stratification through the addition of multiple blood biomarkers in CVD risk prediction modeling. We report the development and validation of risk prediction models for a range of heterogeneous CVDs with different pathogenesis, including coronary artery disease (CAD), stroke, deep venous thrombosis (DVT), and abdominal aortic aneurysm (AAA), as well as for Type 2 diabetes mellitus (DM2), a prime CVD risk factor [19]. The aforementioned diseases together are broadly defined in the present study as cardiometabolic diseases (CMDs). The prediction models were derived using a large population (UK Biobank [20]) analysis with a median longitudinal follow-up of 6.1 years and incorporated a distinct combination of conventional risk factors, blood biomarkers, and comorbidities produced by uncurated variable selection.

## Materials and methods

### Inclusion/exclusion criteria and outcome definition

**Fig 1:**
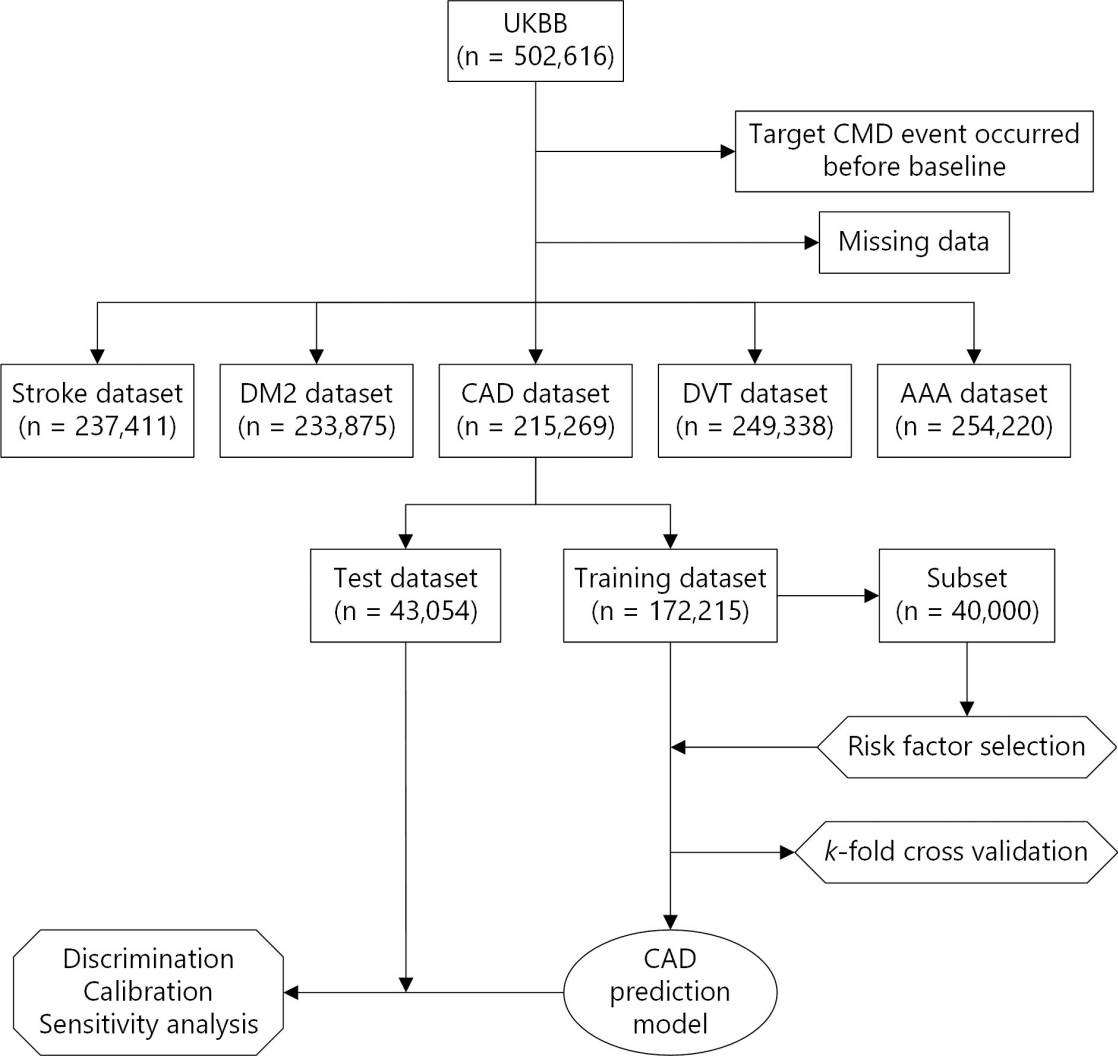
Flow chart demonstrating the exclusion criteria, and the development process of the CAD model, and subsequent validation. The process was the same for the other CMDs.

Baseline data for 502,616 UK Biobank (UKBB) participants collected at assessment centers were used to derive the prediction models. Overall, 95% of the UKBB participants were self-described as white, with women comprising 54.4% of the participant population. The UKBB data was subsequently linked to hospital episode statistics (HES) data from hospitals in England, Scotland, and Wales (**Fig 1**). Outcomes for coronary artery disease (CAD), Type 2 diabetes mellitus (DM2), stroke, deep venous thrombosis (DVT), and abdominal aortic aneurysm (AAA) were determined according to documentation using the following International Classification of Diseases (ICDs) for each of the diseases:

1. International Classification of Diseases edition 10 (ICD-10) codes for all CMD outcomes in the HES data. The following ICDs were used: I20–I25 and T82 codes were used for CAD; E11-E14 codes for DM2; G46.3, G46.4, I63, I66, I67, and I693 codes for stroke; I82, O22.3, R09.89, and Z86.7 codes for DVT; and I71 and I79.0 codes for AAA.
2. Self-reporting for CAD, DM2, and DVT.
3. Self-reported medications for CAD (Nitrolingual, Tildiem) and DM2 (Rosiglitazone, Pioglitazone, Metformin, Isosorbide mononitrate, Insulin products, Glucophage, Glimepiride, Gliclazide).

The age and date of a CMD event were determined based on primary or secondary ICD-10 codes in the HES data corresponding to the earliest hospitalization records. Individuals with more than one CMD diagnosis during a given admission were included in the study samples for corresponding CMD. The date of inclusion in the UKBB was defined as the participant’s baseline and was used as the starting point for time-to-event calculations. Participants with a target CMD event before baseline (identified by ICD-10 codes, self-reports, or medication) were excluded from the study sample for the corresponding CMD modeling (**Fig 1**). However, participants with prior non-target CMD event(s) (potential comorbidity) were not excluded. The above exclusions resulted in five CMD-specific datasets with samples sizes ranging from approximately 466,000 to 481,000. Incident cases were defined as CMD-positive cases per ICD-10 codes and had the date of the event recorded on the HES data after the baseline. Self-reported diagnoses and medications were only used to identify prevalent cases since this information is only available at baseline. Further exclusion of cases due to missing data produced a final five study populations used to develop the prediction models; these had sample sizes of 215,269 (CAD), 233,875 (DM2), 237,411 (stroke), 249,338 (DVT), and 254,220 (AAA) (**Fig 1**). The exit date was determined as the occurrence of either date of death, end of follow-up (February 29, 2016), or a CMD event.

### Risk factors for predictive modeling

Conventional CMD risk factors in the prediction models were selected according to frequency of documentation. Accordingly, the variables selected were missing from less than 80,000 individuals. The list of these risk factors included: age, gender, body mass index (BMI), systolic and diastolic blood pressure (SBP and DBP), physical activity, current and past smoking history, and family history. Physical activity was assessed as the metabolic equivalent of task (MET) and calculated in hours/week according to the "Guidelines for Data Processing and Analysis of the International Physical Activity Questionnaire (IPAQ)” [21]. Family history included mother or father for DM2 and stroke, and mother, father, or siblings for CAD. Binary variables describing these combinations for family history were used in predictive modeling.

Additionally, 22 blood biomarkers, including three blood count tests and 19 biochemical markers, were considered as risk factors. We further considered novel risk factors: self-reported sleep apnea, congestive heart failure, arrhythmia, heart valve problem, irritable bowel syndrome, and hyperthyroidism. The arrhythmia category included atrial fibrillation, atrial flutter, Wolff-Parkinson-White (WPW) syndrome, irregular heartbeat, sick sinus syndrome, and supraventricular tachycardia. The heart valve deficiency category included mitral valve prolapse, mitral stenosis, mitral regurgitation/incompetence, aortic valve disease, aortic stenosis, and aortic regurgitation/incompetence.

### Data preparation and variable selection for predictive modeling

Python 3.6.6 for Windows x64 was used for the preparation of datasets for each CMD according to the approaches described above. The datasets were further split into 80% training and 20% testing sets using the pseudorandom number generator algorithm with a constant initial seed value of 42 (**Fig 1**). Training sets were used for model fitting and assessment and variable selection. Testing sets were solely used for assessing models’ discrimination performance and calibration as well as for sensitivity analysis.

The Recursive Feature Elimination (RFE) method (scikit-learn 0.20.0 Python library) was used to automatically construct the best set of predictor variables for each CMD model from the initially available panel of candidate risk factors. Multiple random forest binary classification models predicting the occurrence of a CMD event by the end of the follow-up period were constructed based on subsets of variables of decreasing size, and the models’ performance was compared [22]. Considering low CMD incidence rate, we used the Balanced Random Forest algorithm (imbalanced-learn 0.4.2 Python library) downsampling majority class to balance it with minority class in a bootstrap sample in each decision tree [23]. The accuracy of classification determined as the fraction of correct predictions was used for the models’ performance evaluation.

The RFE method was combined with a stratified two-fold cross-validation using the following procedure: 1) 40,000 samples were randomly selected from a training set and split into two equal sized subsets with preserved ratio of positive to negative CMD cases; 2) variables were recursively removed one-by-one, and a model using the remaining variables was fitted to one subset, and its accuracy was evaluated on another subset; 3) this process was run once on each subset, and average accuracy was calculated and used for ranking and removing the weakest variables and selecting the best subset of variables. The RFE variable selection was applied to each CMD separately. The gender variable was forced into all CMD-specific sets of explanatory variables.

Additional variable selection based on a variance inflation factor (VIF) detecting correlation between variables was conducted for the DM2 model prior to RFECV to achieve better calibration. *VIF*_*i*_ for each variable was calculated using the following formula:

$${VIF}_{i}=\frac{1}{1-{R_{i}}^{2}},$$

where *R*_*i*_^*2*^ is the coefficient of determination for each variable. *R*_*i*_^*2*^ was calculated by regressing the variable against every other variable using ordinary least squares regression (statsmodels 0.9.0 Python library). Variables with the lowest VIF among all variables with VIF higher than 2 were removed one-by-one until all variables had VIF lower than 2. The VIF variable selection did not improve the calibration when applied to the rest of the CMD models.

### Prediction models and performance metrics

Linear Cox Proportional Hazard (PH) models were developed using lifelines 0.13.0 Python library. Continuous variables were scaled to a range between 0 and 1 to allow for a comparison of the magnitudes of regression coefficients. The discriminative ability of the risk prediction models was assessed by Harrell’s concordance index (C-index) [24–26], which was calculated during the validation and datasets testing as the proportion of all comparable pairs where the predictions and outcomes were concordant. Case pairs were comparable if at least one of them was CMD positive. If the prognostic index was larger for the case with a lower survival time, the prediction of that pair was counted as concordant. If predictions were identical for a pair, 0.5 was added to the count of concordance. A pair was not comparable if an event occurred for both at the same time or an event occurred for one, but the time of censoring was smaller than the time of event of the first one.

*k*-fold cross-validation was used to assess for overfitting leading to model optimism and to adjust estimates of discriminative ability for this optimism [27]. A training set was randomly partitioned into five complementary equal sized subsets. Of the five subsamples, a single subsample was retained as the validation set for testing the discriminative ability of a model, and the remaining four subsets were used as the training set. This process was repeated five times, with each of the five subsets used exactly once as the validation set. The resulting C-indexes were averaged to produce a single, overall optimism-corrected estimate of the C-index with a 95% confidence interval (CI) and standard deviation (SD).

Calibration of Cox PH models was evaluated by the Hosmer-Lemeshow goodness-of-fit test [28]. The Hosmer-Lemeshow test statistic was calculated using the following formula:

$$H=\sum_{g=1}^{G}\frac{({O_{1g}-E_{1g})}^{2}}{N_{g}\pi_{g}(1-\pi_{g})},$$

where *O*_*1g*_ is observed CMD events, *E*_*1g*_ is expected CMD events, *N*_*g*_ is total observations, and *π*_*g*_ is predicted probability for the *g*^th^ risk decile group, and *G* is the number of groups. The testing set was divided into decile groups based on the predicted probability of CMD events for a time horizon of five years. Then, the number of observed CMD events and the sum of the predicted probabilities of CMD events (interpreted as the number of expected CMD events) were calculated in each decile group. The computed Hosmer-Lemeshow statistic was compared to a *chi*-squared distribution with eight (G-2) degrees of freedom to calculate the *P*-value. Calibration was visualized using a calibration plot using the predicted risk probabilities plotted against the observed risks for each decile group.

### Subpopulation sensitivity analysis

The performance and sensitivity of prediction models were assessed in four subgroups of individuals with different age and CMD status. Multiple testing sets were created by applying age and disease filters to the general testing datasets. These subpopulations included (1) “healthy” participants without any of four non-target CMD at the baseline; (2) “unhealthy” participants with one or more pre-existing non-target CMD at the baseline; (3) participants with only one non-target CMD (CAD, DM2, or DVT); and (4) participants in the age categories <45, 45–55, 55–65, and 65–75 years.

## Results

The mean (SD) age at baseline was 56 (8.0) years across all CMD study samples, with women accounting for 54.0% to 55.6% of the participants in these samples. Gender-specific demographics, physiological and lifestyle characteristics, the number of CMD incident events (**Table 1**), as well as biochemical and clinical characteristics (**S1 Table**), showed no significant variation across different CMD study samples. Assessment of the participant’s follow-up (median 6.1 years) reports, the incidence rates for CAD (3.32%), DM2 (2.65%), stroke (0.66%), DVT (2.44%), and AAA (0.17%) were observed in the corresponding study sample.

**Table 1:** Baseline characteristics of the study samples for each dataset used to derive CMD-specific prediction models.

	CAD		DM2		Stroke		DVT		AAA	
	Women	Men	Women	Men	Women	Men	Women	Men	Women	Men
	n = 119,756	n = 95,513	n = 127,929	n = 105,946	n = 129,255	n = 108,156	n = 135,640	n = 115,075	n = 137,352	n = 116,868
Age, mean (SD), y	55 (8)	56 (8)	55 (8)	56 (8)	56 (8)	56 (8)	56 (8)	56 (8)	56 (8)	56 (8)
Body mass index, mean (SD)	26.4 (4.8)	27.3 (4.0)	26.4 (4.8)	27.3 (3.9)	26.5 (4.8)	27.4 (4.0)	26.5 (4.8)	27.4 (4.0)	26.5 (4.8)	27.5 (4.0)
Systolic blood pressure, mean (SD), mm Hg	133 (18)	139 (17)	133 (18.4)	139 (17)	133 (18)	139 (17)	133 (18)	140 (17)	133 (18)	140 (17)
Diastolic blood pressure, mean (SD), mm Hg	80 (10)	84 (10)	80 (10)	84 (10)	80(10)	83 (10)	80 (10)	83 (10)	80 (10)	83 (10)
Forced expiratory volume, mean (SD), %	94.1 (17.7)	93.1 (18.2)	94.1 (17.7)	93.1 (18.3)	94.0 (17.7)	92.8 (18.4)	93.9 (17.8)	92.6 (18.4)	93.9 (17.8)	92.5 (18.5)
Physical activity, mean (SD), MET x hours/week	48.8 (56.8)	59.6 (77.6)	48.7 (56.7)	59.3 (76.9)	48.7 (56.7)	59.0 (76.7)	48.9 (56.3)	59.6 (77.7)	48.9 (57.3)	59.4 (77.6)
Current smoking, No. (%)	9599 (8.02)	10733 (11.2)	10177 (7.96)	11836 (11.2)	10259 (7.94)	12030 (11.1)	11100 (8.18)	13237 (11.5)	11285 (8.22)	13484 (11.5)
Past smoking, No. (%)	65186 (54.4)	60297 (63.1)	69565 (54.4)	67026 (63.3)	70287 (54.4)	68581 (63.4)	74058 (54.6)	73217 (63.6)	75046 (54.6)	74517 (63.8)
Family history, No. (%)	58743 (49.1)	42320 (44.3)	10910 (8.53)	8304 (7.84)	33770 (26.1)	26599 (24.6)	N/A	N/A	N/A	N/A
CMD incident events, No. (%)	2476 (2.07)	4677 (4.90)	2387 (1.87)	3789 (3.58)	617 (0.48)	956 (0.88)	3350 (2.47)	4045 (3.52)	54 (0.039)	378 (0.32)

The discriminative ability of all Cox PH CMD models estimated by five-fold cross-validation varied between the diseases with the highest and lowest C-indexes for DM2 and DVT, respectively. The optimism-corrected estimate of discrimination C-statistic was 0.794 (CI, 0.787–0.801, SD = 0.0050) for CAD, 0.909 (CI, 0.903–0.916, SD = 0.0046) for DM2, 0.778 (CI, 0.756–0.801, SD = 0.0162) for stroke, 0.743 (CI, 0.737–0.749, SD = 0.0044) for DVT, 0.893 (CI, 0.874–0.912, SD = 0.0137) for AAA. A low standard deviation of C-statistic values for the CAD, DM2, and DVT models implied their high reproducibility and good generalization to unknown data from the same population and a low degree of overoptimism (**Table 2**). The models for stroke and AAA, the diseases with lower numbers of incident events, demonstrated a lower reproducibility compared to the other models. Performance assessment in testing sets of the general population (**Table 2**) demonstrated that C-indexes for the CAD and stroke models were within the above 95% CIs. C-indexes for the DM2, DVT, and AAA models were outside of the CIs by 0.003, 0.001, and 0.005, respectively, consistent with the low degree of overoptimism estimated by the cross-validation method.

**Table 2:** Discriminative ability of CMD risk prediction models among different subpopulations.

Test subpopulation	CAD	DM2	Stroke	DVT	AAA
General	0.789 (3.32)	0.9 (2.65)	0.776 (0.66)	0.750 (2.95)	0.869 (0.17)
Healthy + target CMD	0.788 (3.16)	0.903 (2.5)	0.76 (0.57)	0.738 (2.60)	0.874 (0.14)
Unhealthy + target CMD	0.643 (10.8)	0.752 (6.78)	0.65 (2.70)	0.575 (12.4)	0.710 (0.76)
CAD	N/A	0.727 (8.01)	0.663 (2.80)	0.582 (12.3)	0.745 (1.13)
DM2	0.655 (10.9)	N/A	0.646 (3.27)	0.594 (11.3)	N/A
DVT	0.644 (10.9)	0.763 (5.42)	0.633 (3.24)	N/A	N/A
Age < 45	0.741 (0.83)	0.898 (0.8)	0.524 (0.14)	0.645 (0.91)	N/A
Age 45–55	0.742 (1.57)	0.889 (1.51)	0.694 (0.25)	0.677 (1.42)	0.854 (0.03)
Age 55–65	0.734 (3.96)	0.897 (3.03)	0.696 (0.70)	0.705 (3.37)	0.778 (0.17)
Age 65–75	0.724 (7.29)	0.853 (5.19)	0.670 (1.71)	0.656 (6.15)	0.808 (0.51)

Mean C-indexes for CMD models and percent of participants (in parenthesis) that encountered target CMD incident events during the follow-up period.

The number of predictors in the best sets generated for different models varied from nine predictors for AAA to 40 for CAD (**S2 Table**). Among multiple biomarker predictors shared across different models, cystatin C and red blood cell distribution width were common risk factors for all four CVDs, but not for DM2. Comparison of the values of normalized regression coefficients (**S2 Table**) demonstrated that cystatin C was the most crucial risk factor for stroke, DVT, and AAA, and was superseded by glycated hemoglobin only in the CAD model. Glycated hemoglobin also was the most important risk factor for DM2 and was shared among stroke, DVT, and DM2; however, it was not a statistically significant variable for AAA. Overall, the CAD and stroke models shared the largest number of predictors among all diseases.

Broad range applicability and performance consistency for the developed risk prediction models for each disease was further determined by assessing the discriminative ability across subpopulations using sensitivity analysis (**Table 2**). This analysis demonstrated lower prediction in the “unhealthy” compared to the “healthy population,” as defined in the material and methods. The performance of the models was highest in middle age (45–65 years), but it significantly dropped in the subpopulations with pre-existing CMD.

**Fig 2:**
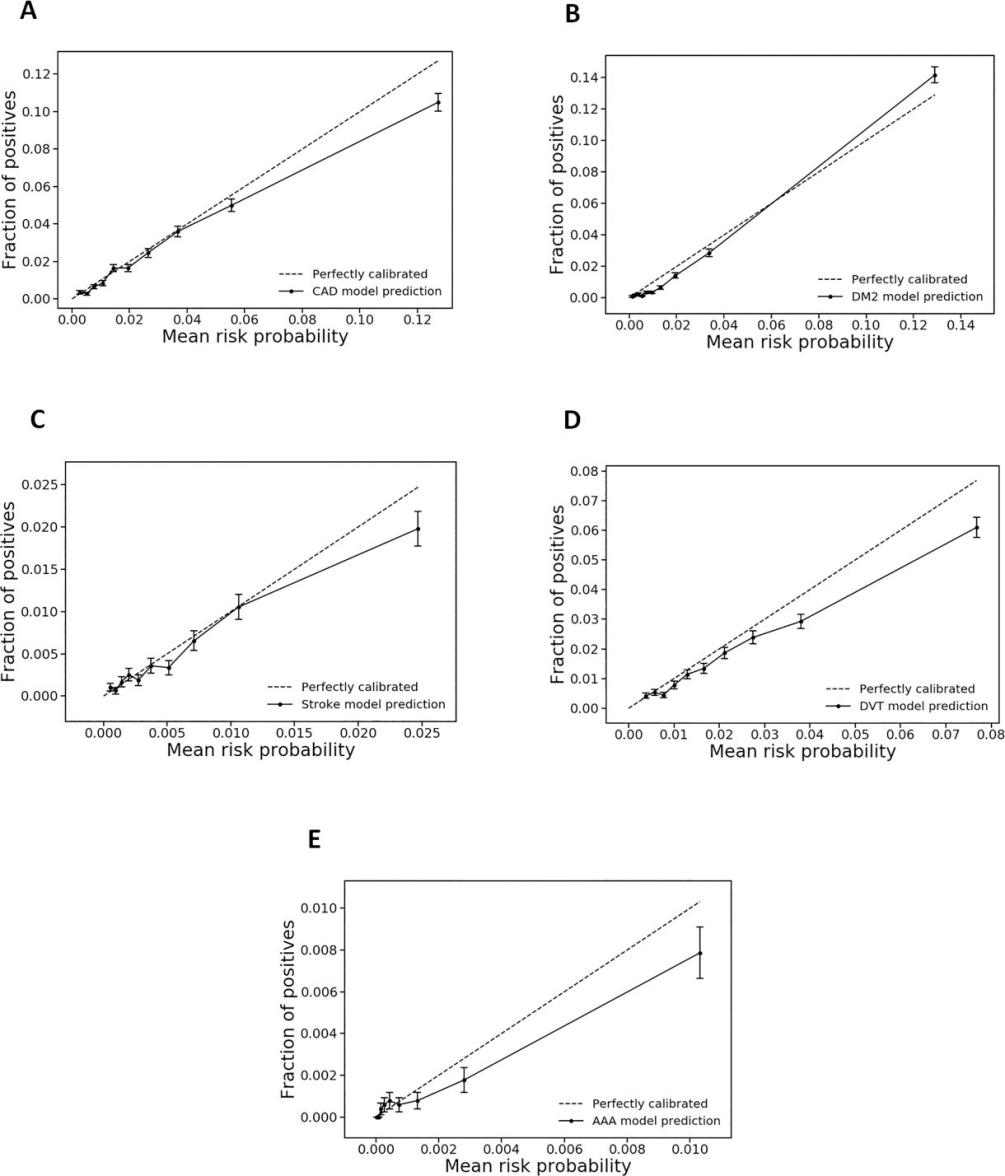
Calibration plots for CMD risk prediction models. Five-year absolute risks for CAD (A), DM2 (B), stroke (C), DVT (D), and AAA (E) were split into deciles, and mean risk probability for each decile was plotted versus the portion of positive CMD cases in the decile for a time horizon of five years.

To evaluate the calibration properties of the prediction models amongst the general population, the five-year absolute risk for each CMD event was calculated. Hosmer-Lemeshow test statistic produced *chi*-squares values (*P*-value) of 33.8 (<0.0001), 77.8 (<0.0001), 12.8 (0.12), 45.3 (<0.0001), and 12.3 (0.14) for the CAD, DM2, stroke, DVT, and AAA models, respectively. Calibration plots (**Fig 2**) demonstrated consistent overall calibration, but overestimation of CMD risk in the highest decile of risk probabilities for all except the DM2 model. DM2 risk was slightly overestimated in the lowest deciles and minimally underestimated in the highest risk decile. As expected, with a low number of events, the prediction model for AAA was poorly calibrated.

**Fig 3:**
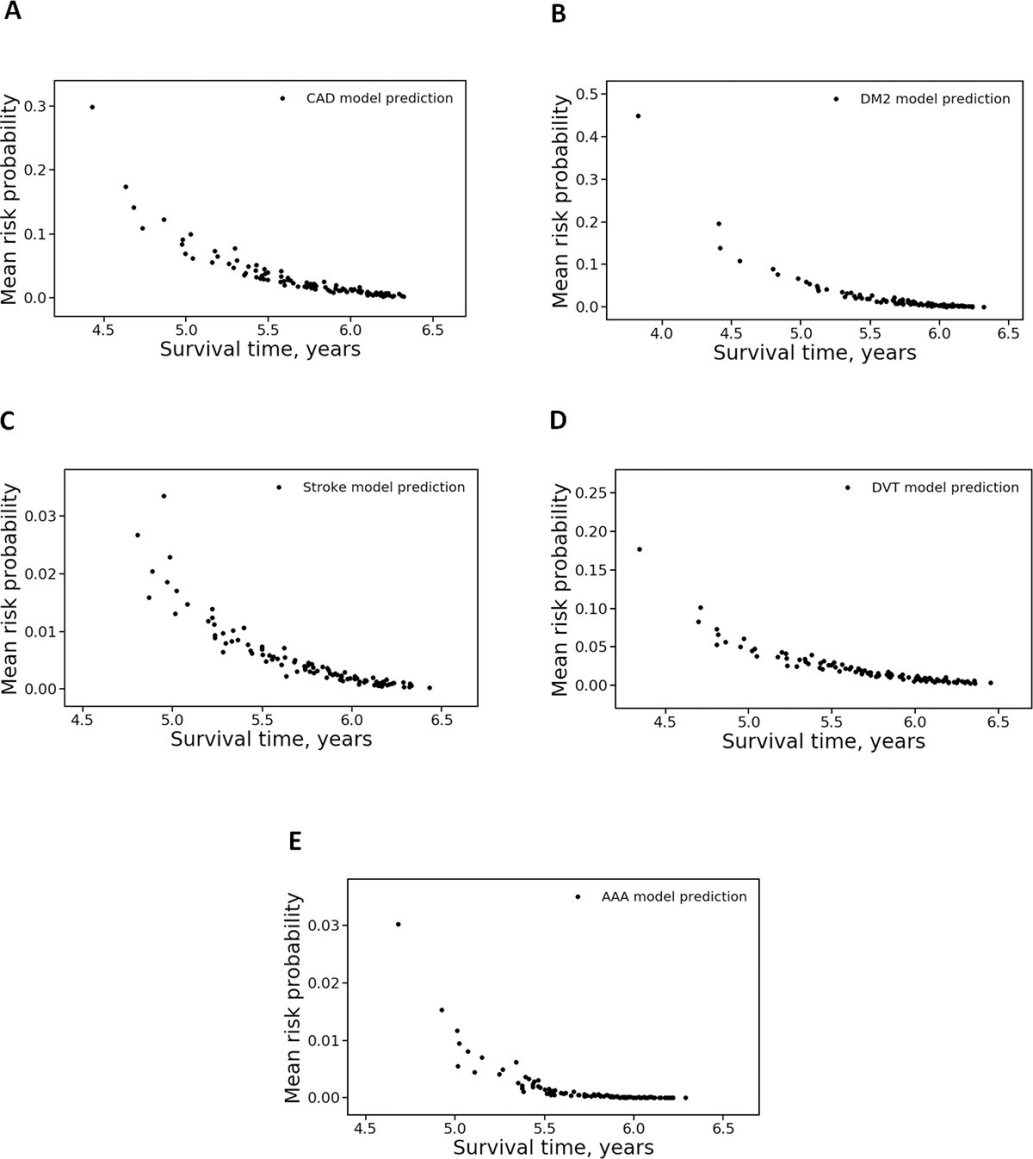
CMD risk prediction models demonstrate the relation between risk level and survival. Risk probabilities for five diseases, CAD (A), DM2 (B), stroke (C), DVT (D), and AAA (E), were split into deciles, and mean risk probability for each decile was plotted versus mean survival time in the decile.

The mean risk probability versus mean survival time for each decile was plotted (**Fig 3**) to evaluate the discriminative ability of individual risk models. The mean risk probabilities for all CMDs exponentially decreased with increased survival time. A steeper exponential decay was observed for the DM2 and AAA models, which demonstrated the highest discriminative ability for these two diseases.

## Discussion

In the present study, the development and validation of risk prediction models for a range of heterogeneous diseases with different pathogenesis, including four CVDs and DM2, were presented. Extensive population data from UK Biobank was applied to produce model-specific training, validation and testing sets. The discriminative ability of individual models for each of the chronic diseases was first examined in the general population. Subsequently, the impact of comorbidities amongst various age groups was determined. The discrimination performances were high in the overall UK Biobank population and remained moderate to high in the subpopulation analysis. Calibration of the five-years survival outcome demonstrated incremental overestimation of disease-related conditions amongst the highest decile of risk probabilities. Internal validation of the developed models demonstrated good reproducibility and a low degree of overoptimism.

In addition to conventional risk factors, the models described in this study incorporated multiple blood biomarkers and comorbidities. Contemporary risk factors such as biomarkers, polygenic risk scores [13, 29–31], and certain metabolomic patterns [32, 33] have been proposed to augment the total risk assessment. As demonstrated previously, depending on the population, up to 50% of patients with CVDs may lack conventional risk factors. However, these conventional risk factors can also fail to identify between 15–50% of those at risk of developing cardiovascular disease [34–38]. Understanding and differentiating between clinical statuses due to acquired risk factors versus genetic predispositions can significantly impact the approaches to risk factors modification, which can change the course of disease progression. Future work will focus on exploring the value of polygenic risk scores to improve the performances of the models described in this report.

The predictive modeling presented also demonstrated that many of the risk factors are shared across various CVDs and DM2, implying complex pathophysiological links. Positive associations reported previously between CAD, stroke, AAA, and DVT, with both cystatin C and red blood cell distribution width [3–8, 10–12, 14], as well as similar, atherosclerosis-based pathogenesis of CAD and stroke also support our observations.

An automatic approach for variable selection developed in this study allowed us to produce disease-specific sets of explanatory variables and to include novel biomarkers that were not previously used in risk stratification for CVDs or DM2. Prediction models for heterogeneous diseases constructed using the same general panel of candidate predictors had a good predictive performance and reproducibility. This is the novelty of our work as compared to previously published risk prediction models for composite CVD (myocardial infarction, angina, coronary heart disease, stroke, and transient ischemic attack) [39], DVT [40], AAA [41] and DM2 [42] that incorporated conventional risk factors selected by a labor-intensive curated process. When applied to large-scale disease-agnostic datasets with a large number of potential predictors derived from electronic health records (EHR), domain knowledge-based variable selection can discard important information. Increasing use of comprehensive EHR for more accurate risk stratification and prediction of patient outcomes [43] further underlines the importance of application of automatic approaches to variable selection.

### Limitations of this study

Given the predominantly white UK Biobank population and the fact that both training and testing datasets were produced from the same population, the developed prediction models are unlikely to be generalizable to other populations, and their transportability requires further assessment in external validation studies. Low transportability is a common limitation of prediction models, including established CVD risk algorithms. It was reported that neither the Framingham (derived from a US cohort [15]) nor ASSIGN (derived from the Scottish Heart Health Extended Cohort [44]) algorithms were well calibrated for the UK population, with both scores tending to over-predict risk [45]. These algorithms also had a decreased discrimination performance in comparison to the QRISK algorithm derived from a large primary care database in England and Wales [45].

Comparison of risk factor associations in UK Biobank against representative, general population a recent study by Batty et al. [46] demonstrated that associations between the risk factors and health outcomes are generalizable. Accordingly, recalibration of risk scores can be performed using these associations and an updated baseline risk for a specific population. It should also be emphasized that even a well-calibrated risk algorithm does not automatically translate to improved patient outcomes. Substantial work is required to make CVD risk stratification a practical and effective clinical tool.

### Future directions

Considering computational limitations of non-linear survival models [47], binary classification models utilizing deep learning algorithms can be adapted in the future to determine the probability of CMD events at certain time horizons. The availability of relatively large healthcare datasets with thousands of potential predictors further supports the application of deep learning in CVD risk assessment. In addition, incorporation of genomic and other omics data may further improve the predictive functionality provided by the developed models.

## Supporting information

S1 Table Baseline biochemical and clinical characteristics of the study samples for each dataset used to derive CMD-specific prediction models. (XLSX)

S2 Table Normalized Cox PH model regression coefficients for five CMDs. Regression coefficients (coef) and corresponding standard errors (se), *P*-values, lower and upper 95% CI are presented.
(XLSX)
